# THE IMPACT OF EARLY CONDITIONS ON THE PREVALENCE OF DIABETES ACROSS THREE COHORTS OF OLDER ADULTS IN MEXICO

**DOI:** 10.1093/geroni/igac059.2130

**Published:** 2022-12-20

**Authors:** Rafael Samper-Ternent, Jesús-Daniel Zazueta-Borboa, Neil Mehta, Cesar González, Rebeca Wong

**Affiliations:** University of Texas Medical Branch, Galveston, Texas, United States; Netherlands Interdisciplinary Demographic Institute, 's-Gravenhage, Zuid-Holland, Netherlands; UTMB, Galveston, Texas, United States; University of COlima, Colima, Colima, Mexico; University of Texas Medical Branch, Galveston, Texas, United States

## Abstract

In low-and-middle-income countries like Mexico the impact of disadvantaged early childhood conditions (ECC) on later-life health in not well known and may be changing across birth cohorts. Our objective is to examine the impact of ECC on the propensity to have diabetes in three different cohorts of older adults aged 60-69 (born in 1930-1939, 1940-1949, 1950-1959). We examine to what extent differences in ECC, sociodemographic characteristics, and lifetime obesity explain the differences in the prevalence of diabetes across the cohorts. We use five waves of data from the Mexican Health and Aging Study (n=10,313). We estimate a series of logistic regression models and use counterfactual analyses to identify the roles of ECC and lifetime obesity on cohort-specific diabetes prevalence and on differences in diabetes prevalence across cohorts. Our results suggest that improvements in ECC would decrease prevalence of diabetes by 12.5% in the 1930-1939 cohort and 10% in the 1950-1959 cohort. However, the rise of obesity at age 50 counters this effect and leads to higher prevalence of diabetes in both cohorts. Our models show that if everyone had a normal weight at age 50, the prevalence of diabetes would decrease by 31.3% for the 1930-1939 cohort, 27.3% for the 1940-1949, and 26.7% for the 1950-1959 cohort. Despite improvements in socio-economic conditions during childhood in Mexico, the increase in lifetime obesity appear to offset the benefits derived from better ECC resulting in higher rates of diabetes.

